# Associations between objectively measured overall and intensity-specific physical activity and phase angle in older adults

**DOI:** 10.1038/s41598-024-57544-7

**Published:** 2024-03-27

**Authors:** Jiaren Chen, Ting-Fu Lai, Chien-Yu Lin, Ming-Chun Hsueh, Jong-Hwan Park, Yung Liao

**Affiliations:** 1 Graduate Institute of Sport, Leisure and Hospitality Management, National Taiwan Normal University, Taipei, Taiwan; 2 Department of Public Health, College of Public Health, China Medical University, Taichung, Taiwan; 3 Faculty of Sport Sciences, Waseda University, Tokorozawa, Japan; 4 Centre for Urban Transitions, Swinburne University of Technology, Melbourne, Australia; 5 Graduate Institute of Sport Pedagogy, University of Taipei, Taipei, Taiwan; 6 Health Convergence Medicine Laboratory, Biomedical Research Institute, Pusan National University Hospital, Busan, South Korea

**Keywords:** Biomarkers, Health care

## Abstract

Phase angle (PhA) is an indicator of cellular health and is positively associated with overall physical activity (PA). However, varied associations between different intensities of PA and PhA by body segment in older populations remain unexplored. We investigated the associations between overall and different intensities of PA and upper-, lower-, and whole-body PhA in older adults. Overall exposure to light-intensity (LPA), moderate-intensity (MPA), and vigorous-intensity physical activity (VPA) was assessed using a triaxial accelerometer (GT3X + , ActiGraph). The outcome variables were upper-, lower-, and whole-body PhA measured using bioelectrical impedance analysis (MC-780MA, TANITA). Multiple linear regression helped examine the associations between the exposure and outcome variables after adjusting for age, gender, body mass index, and accelerometer wear time. A cross-sectional analysis involved 166 community-dwelling older participants (mean age = 72.1 ± 5.5 years; 78.3% women). Overall PA was associated with larger upper- (B: 0.057, 95% confidence interval [CI] 0.018–0.095) and whole-body PhA (B: 0.044, 95% CI 0.006–0.081). LPA was associated with larger upper-body PhA (B: 0.059, 95% CI 0.017–0.101), and MPA was associated with larger lower- (B: 0.273, 95% CI 0.128–0.419) and whole-body PhA (B: 0.141, 95% CI 0.002–0.280). VPA and PhA were not associated. Future interventions targeting PhA in older adults should consider the differential impact of PA intensity on various body segments of the PhA.

## Introduction

The global population of individuals aged above 65 years has been estimated to rise by 120% between 2020 and 2050, from 0.7 to 1.5 billion individuals^1^. In Taiwan, it is estimated that the proportion of the older population will reach 20% by 2025, making it a super-aged society^2^. The age structure shifts toward an older population highlighting the significance of population aging as a notable health concern^3^. The growing population of older adults is associated with heightened susceptibility to age-related ailments, consequently intensifying the burden on healthcare systems^4^.

Well-established evidence underscores the efficacy of engagement in physical activity (PA) as a viable approach for reducing body fat^5^ and fostering muscle mass and enhancing the health of older adults^6,7^. Investigations have indicated inconsistent associations between varying intensities of PA and health outcomes. For example, a previous study of older adults showed that engagement in moderate-intensity physical activity (MPA), but not vigorous-intensity physical activity (VPA), is associated with a higher level of cognitive function^8^. Another study found light-intensity physical activity (LPA) to be beneficially associated with obesity and its markers as well as mortality^9^. Therefore, using overall PA combined with LPA, MPA, and VPA may obscure the differences among these behaviours and their respective roles in enhancing well-being.

Indicators of body composition are of potential interest as they offer insights into assessing well-being in older adults. Adverse body composition, characterized by an excessive accumulation of body fat, elevated levels of visceral fat, and insufficient muscle mass, could cause diseases and disabilities^10^.Previous research has shown direct correlations between fat mass, body mass index (BMI), visceral fat area, and lower levels of PA in older adults^11,12^. There is growing interest in assessing the phase angle (PhA), which is a measure of clinical relevance and a prognostic marker for predicting and monitoring diverse diseases while simultaneously being indicative of the physical state of older adults^13–15^. Defined as the arc tangent of the reactance to resistance ratio (Xc/R), PhA reflects cellular health, with higher values indicating greater cell membrane integrity and tissue vitality^16^. Unlike other body composition metrics, PhA offers a unique insight into its capacity to mirror cell health and nutritional status, exhibiting cellular integrity and metabolic efficiency^17^.

A meta-analysis separately pooling cross-sectional and longitudinal studies across diverse age groups has indicated a favorable impact of engaging in structured exercise on physical function indicated by higher PhA^18^. However, within this body of evidence, the subset of studies that specifically target older adults predominantly investigate the effects of resistance training interventions. So far, two studies examined the association between PA and PhA in older adults^19,20^. One of these studies found that combined MPA and VPA was associated with higher whole-body PhA, without investigating PhA by body segment^19^. Segmental assessment of PhA could potentially serve as an effective means to monitor the health status of specific body parts in older population^21^. Considering the potential variability in the relationship between PA and different body segments^22,23^, it is also recognized that in healthy older adults, disparities exist between the body composition of upper and lower limbs. For instance, lean body mass is associated with upper limb strength, whereas higher body fat has been shown to constrain lower limb performance and endurance^24^. This indicates that a comprehensive assessment cannot be achieved through whole-body PhA measurement alone. Therefore, segmental PhA measurement is crucial to enhance our understanding of how different types and intensities of PA relate to specific body regions, which is essential for developing targeted interventions within public health frameworks.

We hypothesized that there is an association between overall PA and whole-body PhA in Taiwanese older adults, with varying relationships observed between different intensities of PA and segmental PhA. Therefore, this cross-sectional study investigated these associations and conducted a stratified analysis of the PhA according to body segments.

## Methods

### Participants

**Figure 1:**
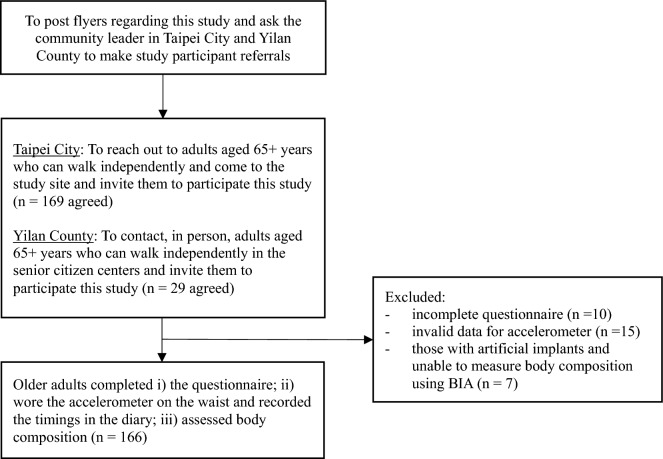
Flow chart of the participant recruitment procedure.

Convenience sampling was used to recruit the study participants. We collaborated with the community leaders in Taipei City and Yilan County, two areas in the northern part of Taiwan, and asked them to refer study participants from May to August 2023 (Fig. 1). Those aged ≥ 65 years who could walk independently were invited, and 198 older adults agreed to participate. They filled in a questionnaire containing items regarding their sociodemographic characteristics (age, gender). They were then asked to wear a tri-axial accelerometer on their waist for seven consecutive days and measure PhA using a bioelectrical impedance analyzer (BIA). Participants who provided missing data on study variables were excluded. Furthermore, the participants with artificial implants (e.g., cardiac pacemakers and joint replacements) were excluded because these implants would impede the measurement of bioelectrical impedance^25^. The final analytic sample was 166 older adults. Each participant provided written informed consent before the survey started, and they received a US$7 gift voucher after filling in the questionnaire, wearing an accelerometer, and assessing PhA using BIA.

### Measurements

#### Phase angle

PhA in degrees (°) was assessed by trained staff using a multifrequency BIA (MC-780MA, TANITA, Tokyo, Japan). The participants were instructed to stand barefoot on the metallic electrode footplates of the analyzer, striated in a neutral standing position, and held on to the metallic handgrips with their arms straight, pointing downwards. Each participant’s five fingers touched the surface of the hand electrode, and their heels and forefeet rested on the circular foot electrode. PhA is an indicator of cellular health, body cell mass, and the integrity of the cell wall^26,27^. It is calculated by dividing the reactance (Xc) by resistance (R) and multiplying the quotient and remainder by 180°/π. A higher PhA value associated with a better physical function^28^. We computed the upper- and lower-body PhAs by averaging the bioimpedance variables of the left and right arms (legs) at a frequency of 50 kHz. Whole-body PhA was determined by summing the upper and lower extremity PhAs, following a methodology outlined in a previous study^29^. The measures have been shown to be reliable and valid for assessing PhA in adults^30^. All procedures were exported using GMON version 3.4.2 (Data Input GmbH, Germany).

#### Physical activity

The duration of LPA (100–2,019 counts/min), MPA (2,020–5,998 counts/min), and VPA (≥ 5,999 counts/min) were assessed using a triaxial accelerometer (ActiGraph GT3X + , Pensacola, FL, USA) on their waist for seven consecutive days^31^. Given that the participants of this study are healthy older adults, and considering that Troiano's cut-points have been adopted in recent research assessing PA levels in this demographic^32–35^, this study has accordingly utilized this cut-point to categorize the varying intensities of PA among the older population. The accelerometer was taken off when they were engaged in water activities (e.g., swimming and showering) only; the time for removing the accelerometer, going to bed, and getting up was recorded in a diary. Overall PA was calculated as the sum of LPA, MPA, and VPA. Data collection and processing criteria for the accelerometer data followed standard protocols^35^. The valid accelerometer data were defined as instances where participants wore the accelerometer i) ≥ 600 min of a single day and ii) at least 4 days (3 weekdays and 1 weekend day). A continuous zero count on the accelerometer for at least 60 min was defined as non-wear time. Data were processed using ActiLife software (version 6.0, Pensacola, FL, USA) with 60-s epochs and a standard sampling frequency of 30 Hz.

#### Covariates

Covariates included age (65–74 years or ≥ 75 years), gender, body mass index, and average daily accelerometer wear time^36,37^. The former two variables were collected using the questionnaire. BMI was calculated by dividing weight (kg) by height (m) squared that retrieved from BIA.

### Statistical analyses

The number and percentage (%) or mean and standard deviation (SD) of the participants’ characteristics were reported. In the multiple linear regression analyses, the independent variables included the overall and different intensities of PA (i.e., LPA, MPA, and VPA). The dependent variables were PhA measurements for the whole-body and for segment-specific (i.e., upper- and lower-body) PhA. Associations between these independent and dependent variables were examined to explore potential relationships. The duration of overall and intensity-specific PA was divided into 30-min units. This segmentation aligns with established physical activity guidelines^38^, ensuring adherence to the recommended daily activity units. Moreover, employing larger time intervals enhances clarity in observing changes in PhA, facilitating interpretation and analysis. Unstandardized coefficients (B) and 95% confidence intervals (CIs) were estimated after adjusting for all covariates. All analyses were performed using IBM SPSS Statistics (version 27.0; IBM Corp., Armonk, NY, 2011). Statistical significance was set at *p* < 0.05. 3.

### Ethics approval and consent to participate

Each participant provided written informed consent before the start of the survey. The Research Ethics Committee of National Taiwan Normal University (REC number: 202112HM024) approved the study protocol. All methods included in this study adhered to the principles of the Helsinki Declaration.

## Results

Table 1 presents the participants’ characteristics. There were 166 participants (78.3% women) with a mean age of 72.1 years (SD = 5.5). Additionally, 72.3% of the study participants were between the ages of 65 and 74. Accelerometer data were collected over seven consecutive days per participant.

**Table 1:** Continuous characteristics of the study participants (n = 166).

Continuous variables	Mean	SD
BMI (kg/m^2^)	23.1	3.3
Light intensity physical activity (min/day)	288.0	71.6
Moderate intensity physical activity (min/day)	21.1	18.7
Vigorous intensity physical activity (min/day)	0.3	1.3
Overall physical activity (min/day)	309.4	77.6
Accelerometer wear time (min/day)	896.9	67.1
The whole-body PhA (°)	5.0°	0.58
The upper-body PhA (°)	5.4°	0.60
The lower-body PhA (°)	4.5°	0.67

BMI: body mass index; SD: standard deviation; PhA: phase angle.

Table 2 shows the associations between PA and PhA. There was a positive association between the overall PA, whole-body, and upper-body PhA, but not with lower-body PhA. When examining intensity-specific PAs, MPA showed a positive relationship with whole-body PhA and lower-body PhA. In contrast, LPA was associated with upper-body PhA. However, no significant association was observed between VPA and PhA in terms of anybody segment.

**Table 2:** Associations of overall physical activity and light-, moderate-, and vigorous-intensity physical activity with whole-, upper-, and lower- body phase angle.

PA	PhA								
	Whole-body			Upper-body			Lower-body		
	B	(95% CI)	*p*	B	(95% CI)	*p*	B	(95% CI)	*p*
Overall	0.044	(0.006, 0.081)	0.023*	0.057	(0.018, 0.095)	0.004*	0.021	(− 0.020, 0.061)	0.322
Age	− 0.170	(− 0.357, 0.018)	0.076	− 0.025	(− 0.219, 0.169)	0.799	− 0.629	(− 0.883, − 0.424)	< 0.001***
Gender	0.408	(0.199, 0.617)	< 0.001***	0.460	(0.244, 0.676)	< 0.001***	0.339	(0.110, 0.567)	0.004**
BMI	0.042	(0.016, 0.067)	0.001**	0.035	(0.009, 0.062)	0.009**	0.046	(0.018, 0.074)	0.001**
Accelerometer wear time	0.000	(− 0.001, 0.001)	0.999	− 0.001	(− 0.002, 0.001)	0.302	0.000	(− 0.001, 0.002)	0.759
Light-intensity	0.039	(− 0.002, 0.079)	0.063	0.059	(0.017, 0.101)	0.006*	0.000	(− 0.044, 0.045)	0.985
Age	− 0.180	(− 0.368, 0.008)	0.060	− 0.036	(− 0.229, 0.158)	0.715	− 0.638	(− 0.843, − 0.434)	< 0.001***
Gender	0.411	(0.200, 0.623)	< 0.001***	0.470	(0.253, 0.687)	< 0.001***	0.328	(0.098, 0.558)	0.005**
BMI	0.040	(0.015, 0.066)	0.002*	0.033	(0.006, 0.059)	0.015*	0.046	(0.018, 0.074)	0.001**
Accelerometer wear time	0.000	(− 0.001, 0.002)	0.845	− 0.001	(− 0.002, 0.001)	0.322	0.001	(− 0.001, 0.002)	0.436
Moderate-intensity	0.141	(0.002, 0.280)	0.046*	0.078	(− 0.068, 0.224)	0.294	0.273	(0.128, 0.419)	< 0.001***
Age	− 0.163	(− 0.352, 0.026)	0.091	− 0.037	(− 0.236, 0.162)	0.715	− 0.585	(− 0.783, − 0.387)	< 0.001***
Gender	0.365	(0.155, 0.575)	0.001**	0.419	(0.198, 0.640)	< 0.001***	0.288	(0.068, 0.508)	0.011*
BMI	0.048	(0.022, 0.074)	< 0.001***	0.039	(0.011, 0.066)	0.006**	0.057	(0.030, 0.084)	< 0.001***
Accelerometer wear time	0.001	(− 0.001, 0.002)	0.324	0.000	(− 0.001, 0.002)	0.783	0.000	(− 0.001, 0.002)	0.681
Vigorous-intensity	0.535	(− 1.385, 2.455)	0.583	0.859	(− 1.140, 2.857)	0.397	0.433	(− 1.636, 2.502)	0.680
Age	− 0.186	(− 0.376, 0.004)	0.055	− 0.045	(− 0.243, 0.153)	0.656	− 0.635	(− 0.840, − 0.430)	< 0.001***
Gender	0.383	(0.171, 0.595)	< 0.001***	0.426	(0.206, 0.646)	< 0.001***	0.326	(0.098, 0.554)	0.005**
BMI	0.043	(0.017, 0.069)	0.001**	0.037	(0.010, 0.064)	0.008**	0.047	(0.019, 0.075)	0.001**
Accelerometer wear time	0.001	(0.000. 0.002)	0.213	0.000	(− 0.001, 0.002)	0.692	0.001	(− 0.001, 0.002)	0.373

B: unstandardized coefficient; CI: confidence interval; PA: physical activity; PhA: phase angle.

The model adjusted for age, gender, BMI, and accelerometer wear time.

*p < 0.05. ***p < 0.001.

## Discussion

Overall PA was associated with larger whole-body and upper-body PhA, which may indicate a tendency towards better body composition, among older adults. Positive associations between different intensities of PA and PhA of different body segments were observed: MPA and whole-body PhA, and lower-body PhA, LPA, and upper-body PhA.

We found a positive association between overall PA and whole-body PhA among older adults, which is similar to previous findings^20^. For every additional 30 min of overall PA, the whole-body PhA increased by 0.044 degrees. This relationship may be partly explained by the fact that a higher volume of regular PA is related to decreased levels of interleukin-6 (IL-6) and increased levels of interleukin-10 (IL-10) in older adults^39^, factors that correlate with higher PhA values^40^.

Positive associations between MPA and whole-body and lower-body PhA were found. For every additional 30 min of MPA, the whole-body PhA increased by 0.141 degrees, and the lower-body PhA increased by 0.273 degrees. While there have been no studies to directly examined these associations and thus the specific mechanisms remain unclear, our findings are in line with previous research^19,41^ that has demonstrated a significant positive association between MVPA, resistance training, and higher PhA. This association may be attributed in part to the fact that the majority of MVPA and exercise activities undertaken by older adults are of moderate intensity. Furthermore, another possible explanation is that PhA serves as an indicator of cell health^40^, evidence confirmed that MPA, in comparison to both LPA and VPA, can provide a protective effect on peripheral blood mononuclear cells telomere length^42^. This protective effect helps to maintain telomere length, delaying senescence, and tissue aging, thereby forestalling cellular aging that indicated by PhA^42,43^. One potential explanation for the specific association between MPA and lower-body PhA may be related to the nature of MPA itself. MPA typically involves activities that heavily engage the lower limb strength, such as brisk walking and stair climbing^44^. However, no significant association was found between VPA and PhA, which might be attributed to the minimal proportion of VPA in the total physical activity time of the study participants (0.096%), averaging only 0.3 min per day.

The duration of LPA showed a positive association with upper-body PhA only. For every additional 30 min of light-intensity physical activity, the upper-body phase angle increased by 0.059, a relationship not found in prior studies. Therefore, the specific physiological pathways underlying these observations have not been definitively established. The findings of two previous Japanese studies^19,45^ have examined no significant relationship between LPA and PhA. This inconsistency may be attributed to the differing extent of LPA in our study compared to previous studies. Additionally, in older individuals, LPA often involves tasks, such as cooking or laundry^44^, which predominantly involve the upper limbs.

To the best of our knowledge, this is the first study to examine the association of overall and intensity-specific PA with upper-, lower-, and whole-body PhA in an older population. The strength of this study includes both PA and PhA were measured objectively. This study has some limitations. The association between PA and PhA could not be inferred due to the cross-sectional design. Future studies employing a longitudinal design are needed to verify these causal relationships. Accelerometer-based PA did not include water-related activities, such as swimming, which may underestimate the PA duration, particularly for MPA and VPA. Furthermore, the cut points for older populations are not yet standardized, which may lead to variations in the classification of LPA, MPA, and VPA^35,46^. Future research should accumulate more evidence to verify their relationship with PhA in older adults. Other residual confounders may affect the association between PA and PhA, such as dietary intake^47^. Future investigations warrant to collect detailed information to reduce the potentially residual confounding. Additionally, future studies could benefit from employing compositional data analysis^48,49^ to account for the co-dependent nature of daily movement behaviors and more accurately assess the interplay between physical activity, sedentary behavior, and sleep.

## Conclusion

There are positive associations between the overall PA and whole-body PhA in older adults, particularly in cases of MPA. Future strategies or interventions aimed at increasing older adults’ engagement in daily MPA, such as brisk walking and stair climbing, should be encouraged for their potential positive association with body composition and well-being.

## Data Availability

The data used in this study can be obtained from the corresponding author upon request.
